# Supplementary figures and images for: HilE mediates motility thermoregulation in typhoidal Salmonella serovars at elevated physiological temperatures
Source: PLoS Pathog. 2025 Oct 16;21(10):e1013133. doi: 10.1371/journal.ppat.1013133 (PMC12561990; doi:10.1371/journal.ppat.1013133)

**A**

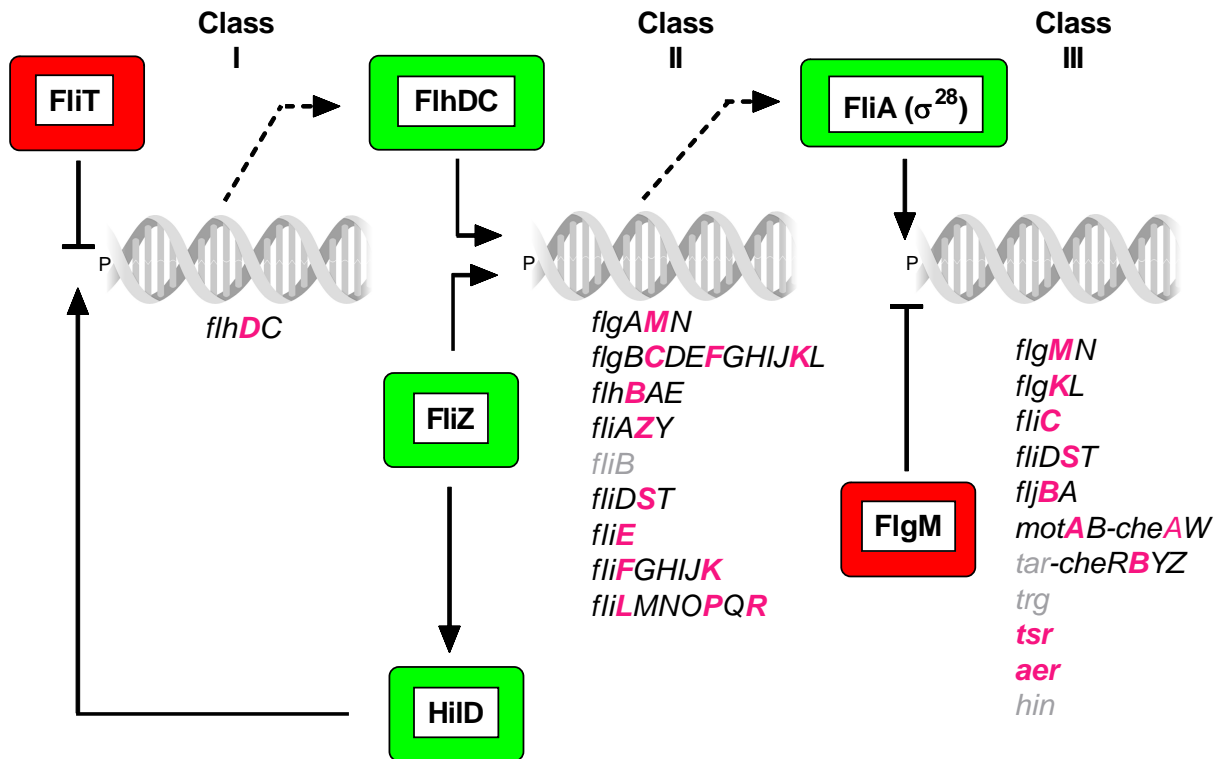

# B

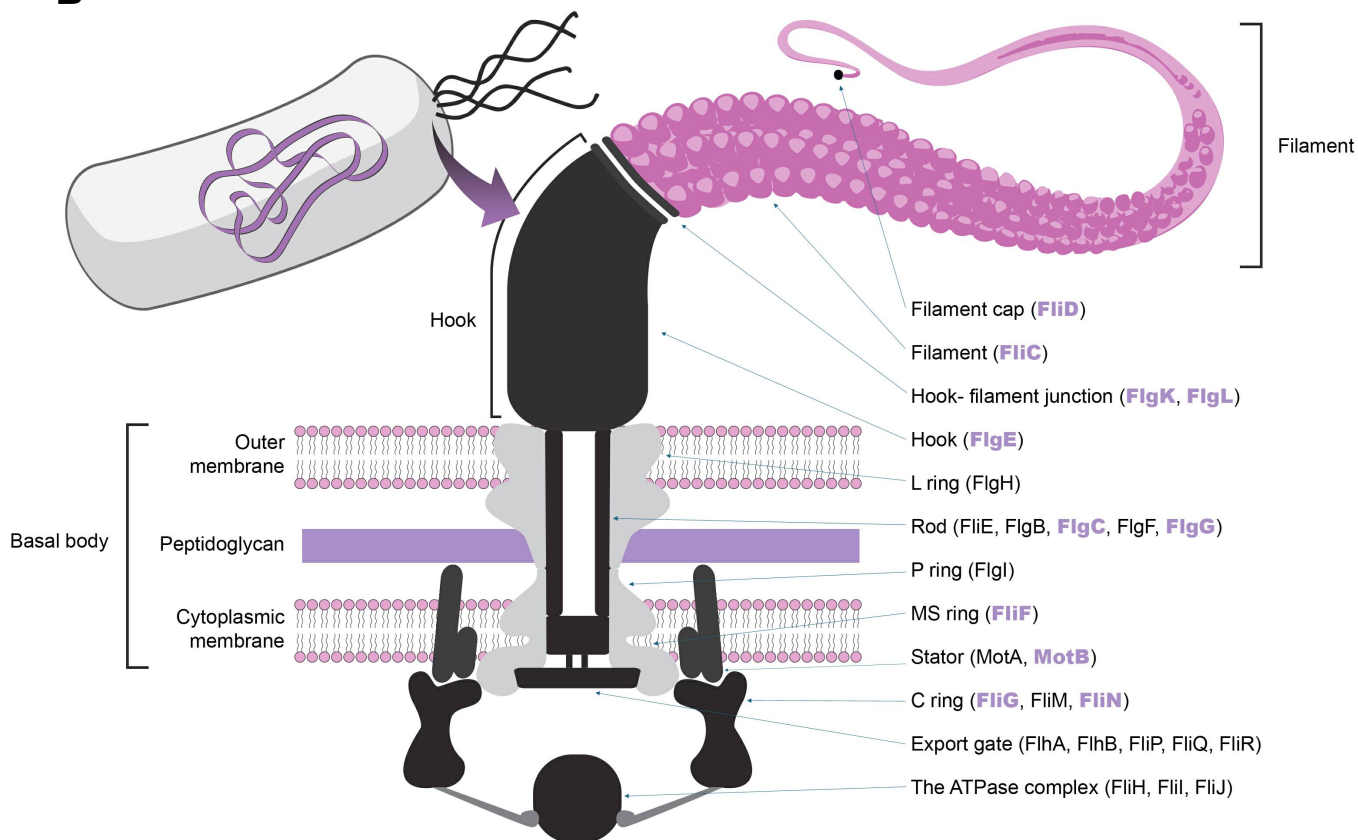

**Fig. S1**

Supplement: S1 Fig — (A) The motility-chemotaxis regulon in Salmonella is organized in three hierarchical transcriptional classes. The class 1 operon contains the flhD and flhC genes that together encode the master regulator of flagella-chemotaxis regulon, FlhDC. The heteromultimeric complex (FlhD4C2) is positioned at the top of this hierarchy and activates the transcription of class 2 operons. Class 2 genes responsible for basal body assembly. Following the assembly of the early flagellar basal body, the repressor FlgM is exported from the cell, releasing the class 3 transcription factor σ28 (FliA). This activates the expression of class 3 genes, leading to the completion of the hook and filament assembly. The positive regulators HilD, FliZ, FlhDC and FliA are shown in green, while the negative regulators involved FliT and FlgM are shown in red. Motility genes that were analyzed by RT-qPCR in Fig. 1A are shown in magenta. Genes that are inactivated in S. Paratyphi A are shown in light grey. (B) Graphical illustration of the flagella structure. Proteins that were detected by the MS analysis in Fig. 1B are shown in purple. (PDF) [file ppat.1013133.s001.pdf]

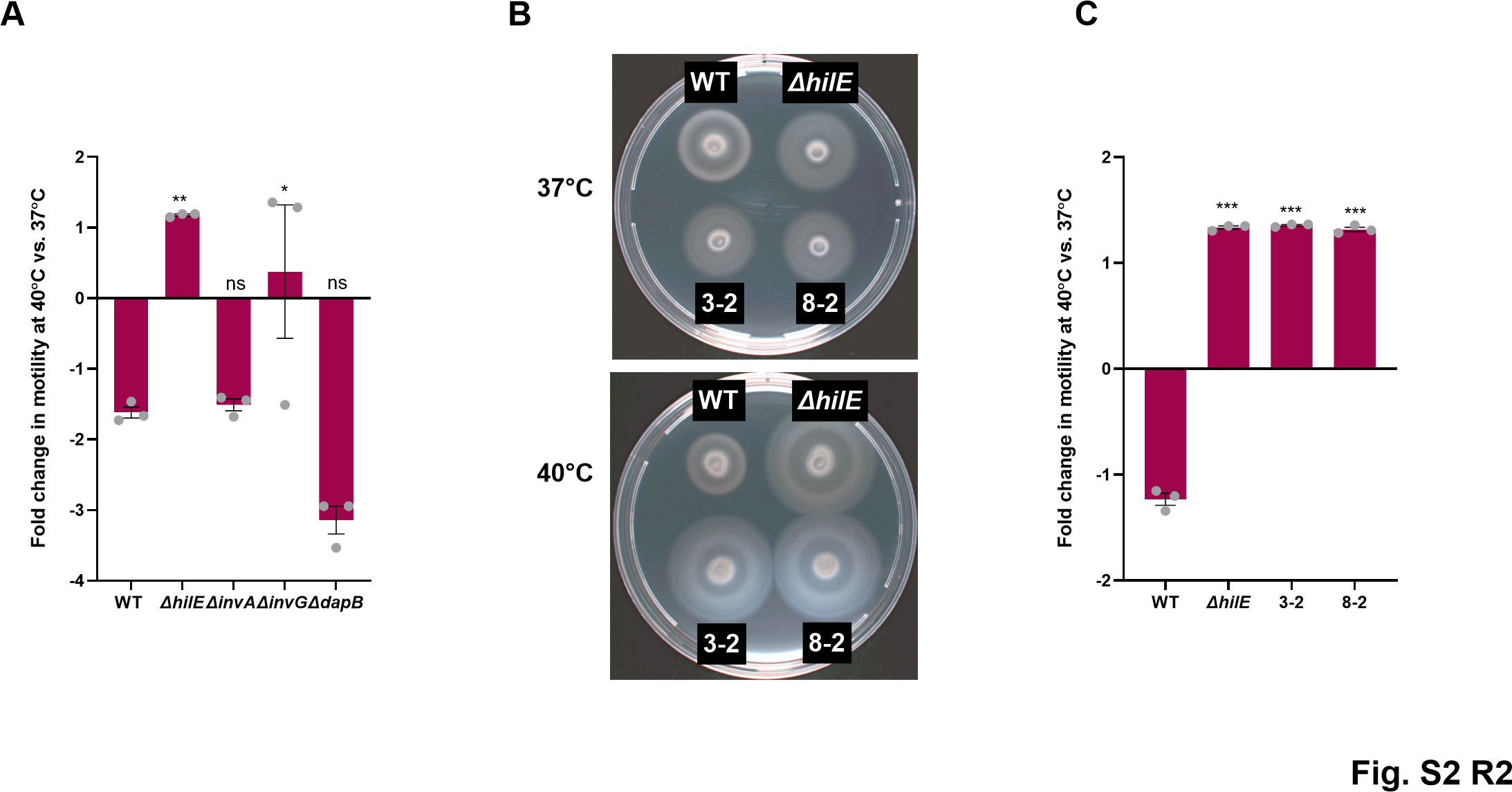

Supplement: S2 Fig — (A) S. Paratyphi A WT and its isogenic null mutant strains ΔhilE, ΔinvG, ΔinvA and ΔdapD were grown overnight at 37°C, spotted onto soft agar plates and incubated at 37°C and 40°C for 5.5 h. The fold change motility at 40°C vs. 37°C is shown, while SEM is represented by the error bars. One-way ANOVA was used to determine statistical significance in relation to S. Paratyphi A WT. (B) Swimming motility on soft agar plates of S. Paratyphi A WT, its isogenic ΔhilE strain and two transposon insertion mutants in hilE (clones 3–2 and 8–2). All strains were grown overnight at 37°C and then 10 µl from each culture were spotted on soft agar LB plates that were incubated at 37°C and 40°C for 5.5 h. (C) The motility radius of the strains was measured and their fold change in motility at 40°C vs. 37°C is shown. One-way ANOVA was used to determine statistical significance relative to S. Paratyphi A WT. *, P-value <0.05; **, P-value <0.01; ***, P-value < 0.001; ns, not statistically significant. (TIF) [file ppat.1013133.s002.tif]

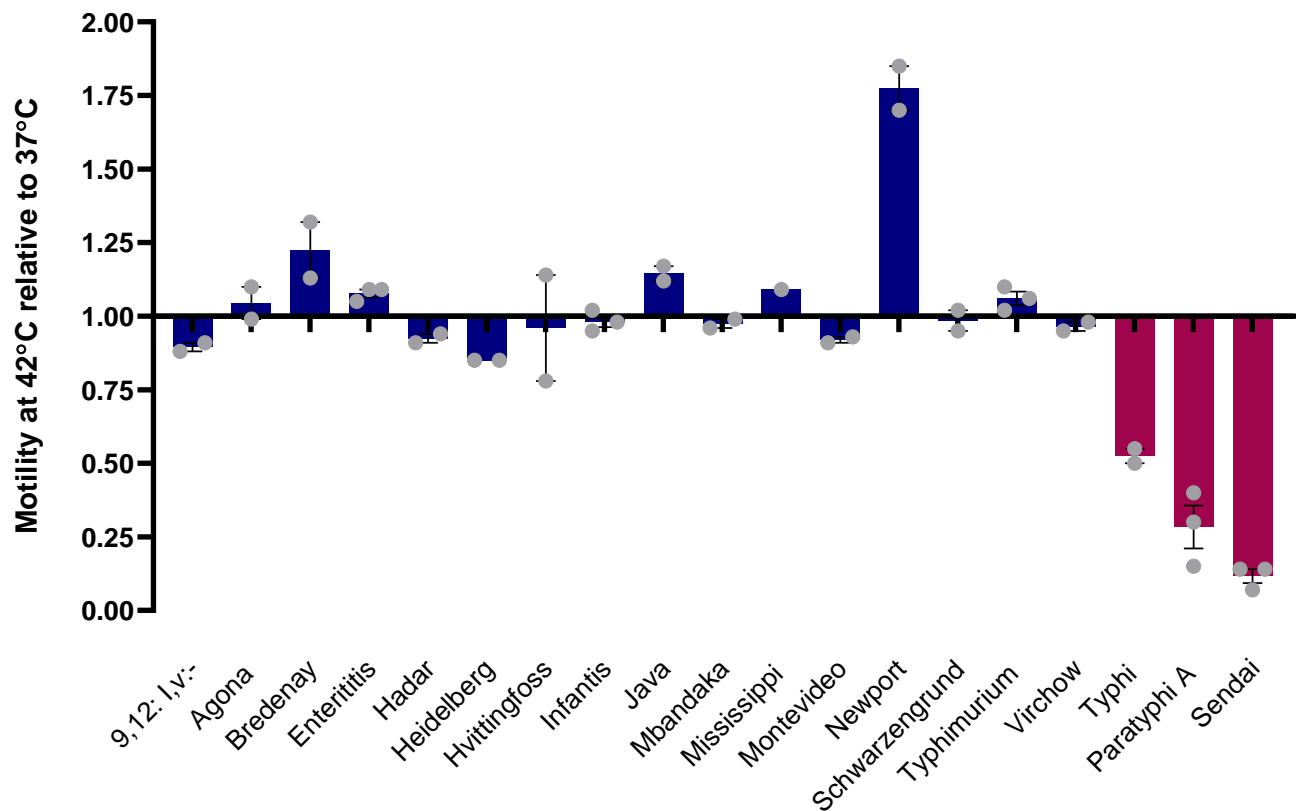

**Fig. S3**

Supplement: S3 Fig — The motility of three typhoidal (Typhi, Paratyphi A, and Sendai) and 16 NTS serovars was measured on semisolid LB agar plates at 37°C and 42°C. The mean change in the motility between 42°C and 37°C is shown. Bars represent the mean of 2–3 independent experiments and SEM is indicated by the error bars. (PDF) [file ppat.1013133.s003.pdf]

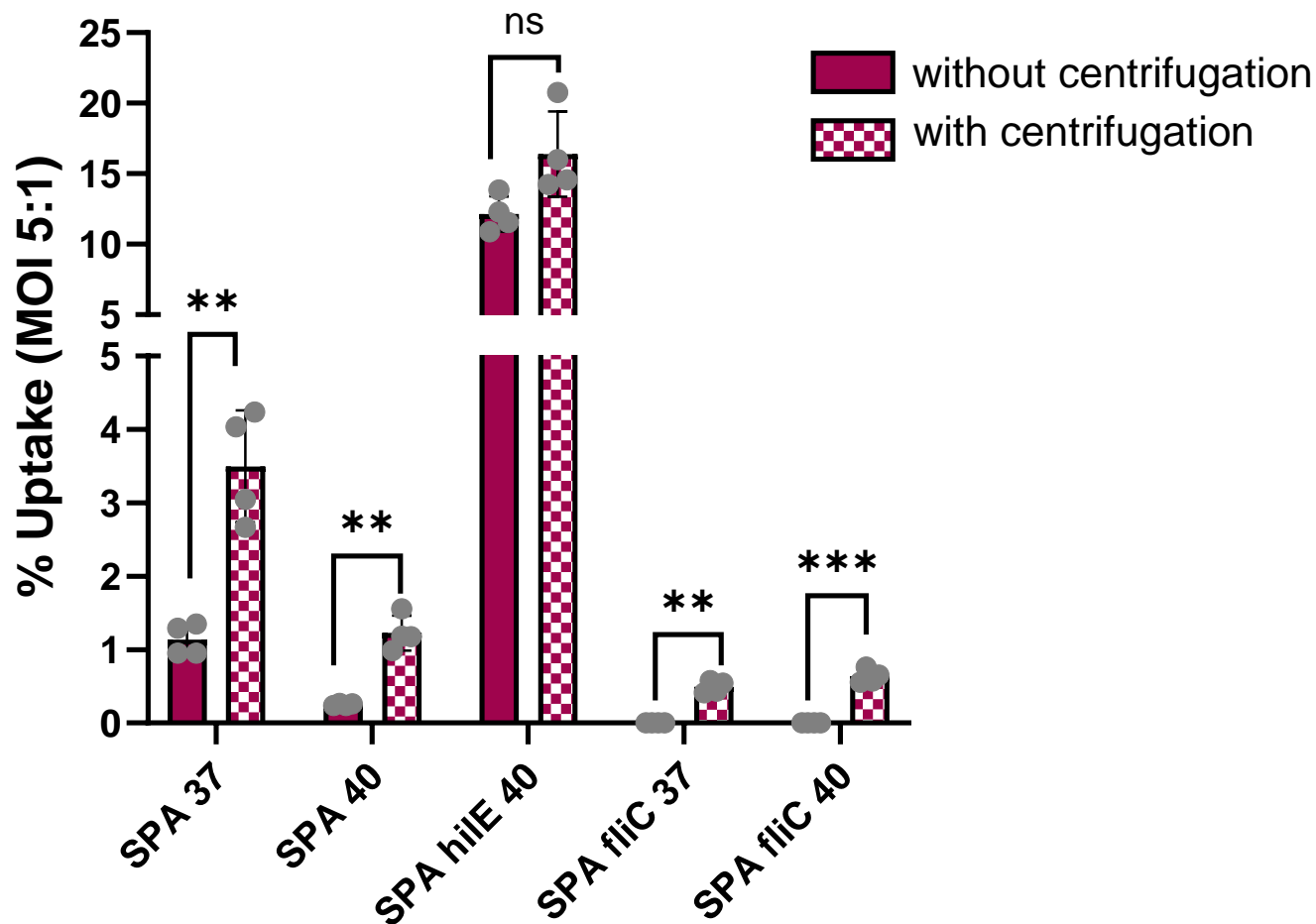

Fig. S4

Supplement: S4 Fig — Cultures of S. Paratyphi A (WT and its isogenic hilE and fliC mutants) were grown at 37 or 40°C and were used to infect differentiated THP-1 human macrophage-like cells at MOI of five at both temperatures. Salmonella uptake by THP-1 cells was determined using the gentamicin protection assay and calculated the percentage of the intracellular CFUs recovered at 2 h p.i from the total number of CFUs used to infect the cells. Bars present the mean value four biological repeats and thier calculated SEM. Unpaired, 2-tailed Student t test was used to calculate statistical significance. (PDF) [file ppat.1013133.s004.pdf]

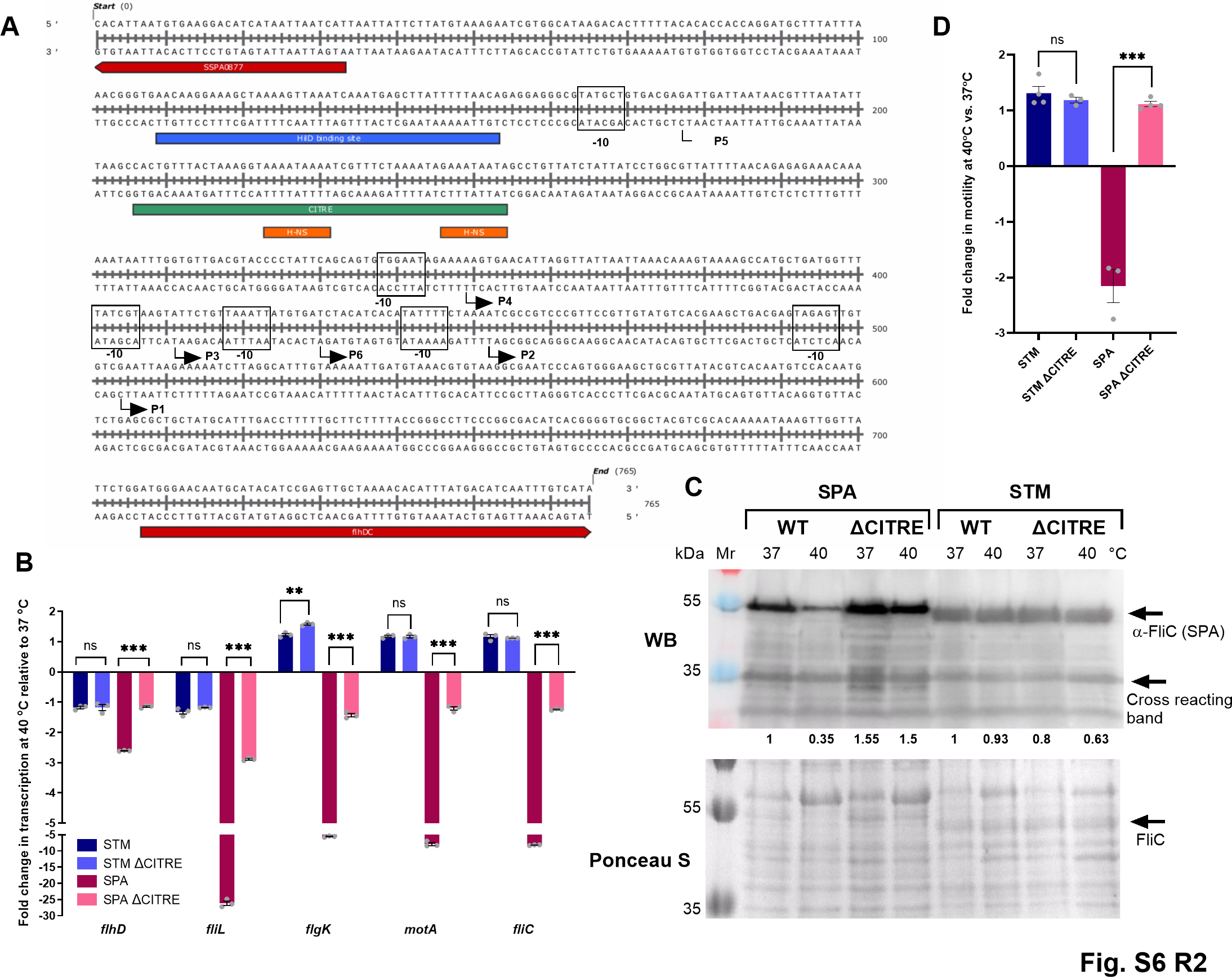

Supplement: S6 Fig — (A) The genetic organization of the flhDC promotor is shown. HilD binding site is presented according to Singer et al. [17]. Black arrows indicate the six flhDC transcription start sites, while their -10 promoter elements are indicated by a closed box, as reported by Yanagihara et al. [58]. The HilD binding site is highlighted in blue and the identified 49-bp CITRE element is marked by a green bar, overlapping with two putative H-NS binding sites predicted by PRODORIC (https://www.prodoric.de) that are shown as orange boxes. (B) WT S. Typhimurium, WT S. Paratyphi A, and their isogenic strains harboring a 49-bp deletion of the CITRE element (∆CITRE) were subcultured in LB grown for 3 h at 37°C and 40°C and subjected to RT-qPCR analysis. Fold change in the expression of five flagellar genes (flhD, fliL, flgK, motA, and fliC) at 40°C relative to their expression at 37°C is shown as the mean value of three biological replicates, with SEM represented by the error bars. Unpaired, 2-tailed Student t test was used to determine statistical significance between the WT background and its isogenic ∆CITRE mutant. (C) The level of FliC at the cellular fraction was analyzed by Western blotting using a S. Paratyphi A anti-FliC antibody. Ponceau S staining and the detection of a ~ 34 kDa cross-reacting band were used as loading controls. FliC expression in the ∆CITRE strain, relative to its expression in the WT background at 37°C, is presented by numerical values below the WB. (D) Cultures of the above strains were spotted onto soft agar plates that were incubated at 37 and 40°C, for 5.5 h. The fold change in motility at 40°C relative to 37°C is shown, while SEM is represented by the error bars. Unpaired, 2-tailed Student t test was used to determine statistical significance. **, P-value <0.01; ***, P-value < 0.001; ns, not statistically significant. (TIF) [file ppat.1013133.s006.tif]
