# Supplementary material for: HilE mediates motility thermoregulation in typhoidal Salmonella serovars at elevated physiological temperatures
Source: PLoS Pathog. 2025 Oct 16;21(10):e1013133. doi: 10.1371/journal.ppat.1013133 (PMC12561990; doi:10.1371/journal.ppat.1013133)
Supplement: S5 Fig — (A) The 310 amino acid protein sequence of HilD in S. Typhimurium strain SL1344 (accession number CBW18953.1) and its ortholog in S. Paratyphi A strain 45157 (accession number QWV88109.1) were aligned using CLUSTALW. Alignment image of the amino acid identity was created by the BoxShade tool (https://junli.netlify.app/apps/boxshade/) and show complete identity at the protein level between these two orthologues. (B) 177 amino acids of the HilE protein in S. Typhimurium strain SL1344 (accession number CBW20528.1) and its ortholog in S. Paratyphi A strain 45157 (accession number QWV89528.1) were aligned and presented as above. Three divergent amino acids between these serovars are highlighted. (C) S. Typhimurium strains including WT, ∆hilE, ∆hilE complemented with the empty vector (∆hilE/P), hilE from S. Typhimurium (∆hilE/hilESTM) or hilE from S. Paratyphi A (∆hilE/hilESPA); and S. Paratyphi A strains including WT, ∆hilE, and ∆hilE complemented with the empty vector (∆hilE/P), hilE from S. Paratyphi A (∆hilE/hilESPA) or S. Typhimurium (∆hilE/hilESTM) were grown at 37°C aerobically for overnight. Ten µl from each culture were spotted on soft LB agar plates that were incubated at 37°C or 40°C for 4 h before imaging. (PDF) [file ppat.1013133.s005.pdf]

A

```
                    1 ........10........20........30........40........50........60........70........80
HilD_STM_SL1344     1 MENVTFVSNSHQRPAADNLQKLKSLLTNTRQQIKSQTQQVTIKNLYVSSFTLVCFRSGKLTISNNHDTIYCDEPGMLVLK
HilD_SPA_45157      1 MENVTFVSNSHQRPAADNLQKLKSLLTNTRQQIKSQTQQVTIKNLYVSSFTLVCFRSGKLTISNNHDTIYCDEPGMLVLK
consensus           1 MENVTFVSNSHQRPAADNLQKLKSLLTNTRQQIKSQTQQVTIKNLYVSSFTLVCFRSGKLTISNNHDTIYCDEPGMLVLK

                   81 ........90.......100.......110.......120.......130.......140.......150.......160
HilD_STM_SL1344    81 KEQVVNVTLEEVNGHMDFDILEIPTQRLGALYALIPNEQQTKMAVPTEKAQKIFYTPDFPARREVFEHLKTAFSCTKDTS
HilD_SPA_45157    81 KEQVVNVTLEEVNGHMDFDILEIPTQRLGALYALIPNEQQTKMAVPTEKAQKIFYTPDFPARREVFEHLKTAFSCTKDTS
consensus          81 KEQVVNVTLEEVNGHMDFDILEIPTQRLGALYALIPNEQQTKMAVPTEKAQKIFYTPDFPARREVFEHLKTAFSCTKDTS

                  161 .......170.......180.......190.......200.......210.......220.......230.......240
HilD_STM_SL1344   161 KGCSNCNNKSCIENEELIPYFLLFLLTAFLRLPESYEIILSSAQITLKERVYNIISSSPSRQWKLTDVADHIFMSTSTLK
HilD_SPA_45157    161 KGCSNCNNKSCIENEELIPYFLLFLLTAFLRLPESYEIILSSAQITLKERVYNIISSSPSRQWKLTDVADHIFMSTSTLK
consensus         161 KGCSNCNNKSCIENEELIPYFLLFLLTAFLRLPESYEIILSSAQITLKERVYNIISSSPSRQWKLTDVADHIFMSTSTLK

                  241 .......250.......260.......270.......280.......290.......300.........
HilD_STM_SL1344   241 RKLAEEGTSFSDIYLSARMNQAAKLLRIGNHNVNAVALKCGYDSTSYFIQCFKKYFKTTPSTFIKMANH
HilD_SPA_45157    241 RKLAEEGTSFSDIYLSARMNQAAKLLRIGNHNVNAVALKCGYDSTSYFIQCFKKYFKTTPSTFIKMANH
consensus         241 RKLAEEGTSFSDIYLSARMNQAAKLLRIGNHNVNAVALKCGYDSTSYFIQCFKKYFKTTPSTFIKMANH
```

B

```
                    1 ........10........20........30........40........50........60........70........80
HilE_STM_SL1344     1 MAGKWNVLSLLAFRPCQPALPHDNHGGGMDAIYLKLDGIEGESRIKGFENQIKLIAYNHNPTKRESGEARGTYIGGLTLT
HilE_SPA_45157      1 MAGKWNVLSLLAFRPCQPALPHDNHGGGMDAIYLKLDGIEGESQIKGFENQIKLIAYNHNPTKRESGEARGTYIGGLTLT
consensus           1 MAGKWNVLSLLAFRPCQPALPHDNHGGGMDAIYLKLDGIEGES IKGFENQIKLIAYNHNPTKRESGEARGTYIGGLTLT

                   81 ........90.......100.......110.......120.......130.......140.......150.......160
HilE_STM_SL1344    81 KPVDLATPGLYEHYCNGKTVKEGVLTLCRRDKGAMLPFIIYTLTNVRISRMSNHGDAEGSATETVDLVYSHIRWDIPALA
HilE_SPA_45157     81 KPVDLATPGLYEHYCNGKTVKEGVLTLCRSDKGAMLPFIIYTLTNVRISRMSNHGDAEGSAMETVDLVYSHIRWDIPALA
consensus          81 KPVDLATPGLYEHYCNGKTVKEGVLTLCR DKGAMLPFIIYTLTNVRISRMSNHGDAEGSA ETVDLVYSHIRWDIPALA

                  161 .......170......
HilE_STM_SL1344   161 SKSKTRRPLHRQALWR
HilE_SPA_45157    161 SKSKTRRPLHRQALWR
consensus         161 SKSKTRRPLHRQALWR
```

C

STM SPA

37°C

40°C

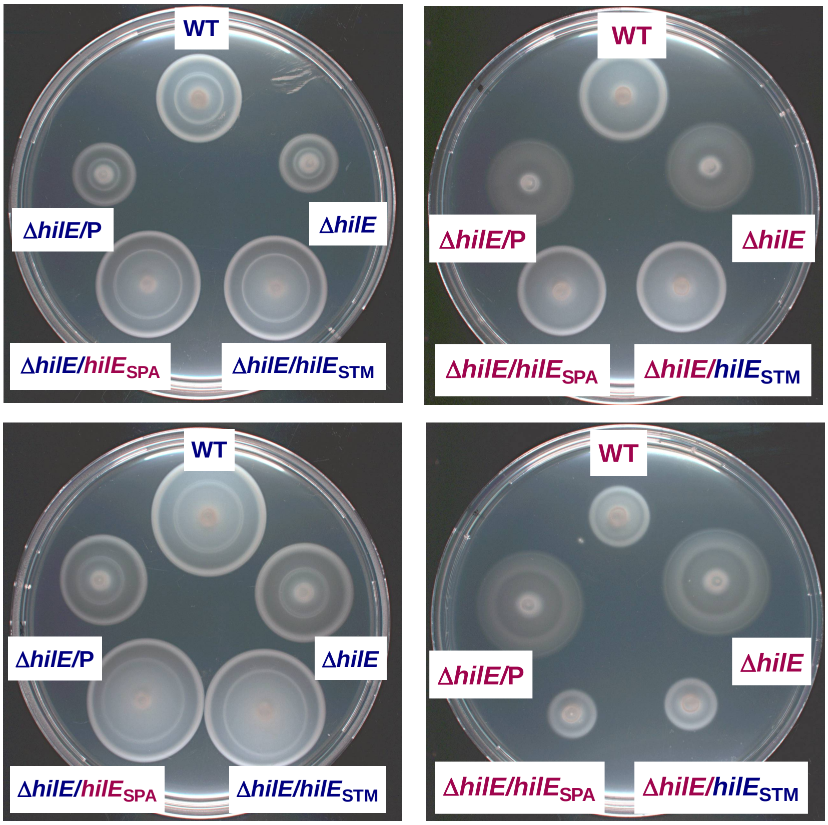

Fig. S5
