# Supplementary material for: HilE mediates motility thermoregulation in typhoidal Salmonella serovars at elevated physiological temperatures
Source: PLoS Pathog. 2025 Oct 16;21(10):e1013133. doi: 10.1371/journal.ppat.1013133 (PMC12561990; doi:10.1371/journal.ppat.1013133)
Supplement: S1 Table — (DOCX) [file ppat.1013133.s007.docx]

**Table S1. Bacterial strains and plasmids used in this study**.

| **Bacterial strain or plasmid** | **Description** | **Reference or source** |
| --- | --- | --- |
| *Salmonella enterica* strains | | |
| *S.* Paratyphi A 45157 | 2009 Nepal outbreak strain *S*. Paratyphi A | [1] |
| *S*. Paratyphi A Δ*hilE* | Null deletion of *hilE* in *S.* Paratyphi A 45157 | This study |
| *S*. Paratyphi A Δ*dapB* | Null deletion of *dapB* in *S.* Paratyphi A 45157 | This study |
| *S*. Paratyphi A Δ*invA* | Null deletion of *invA* in *S.* Paratyphi A 45157 | [2] |
| *S*. Paratyphi A Δ*invG* | Null deletion of *invG* in *S.* Paratyphi A 45157 | [2] |
| *S*. Paratyphi A Δ*fliC* | Null deletion of *fliC* in *S.* Paratyphi A 45157 | This study |
| *S*. Paratyphi A Δ*fliC*Δ*hilE* | *S.* Paratyphi 45157 missing *fliC* and *hilE* genes | This study |
| *S*. Paratyphi A ∆CITRE | *S*. Paratyphi 45157 missing -453 to -501 at *flhDC* promotor | This study |
| *S.* Typhimurium SL1344 | wild type Sm^r^ *xyl hisG rpsL* | [3] |
| *S*. Typhimurium Δ*hilE* | Null deletion of *hilE* in *S*. Typhimurium SL1344 | This study |
| *S*. Typhimurium Δ*fliC* | *S*. Typhimurium SL1344 missing *fliC* gene | [4] |
| *S*. Typhimurium ∆CITRE | *S*. Typhimurium SL1344 missing -453 to -501 at *flhDC* promotor position | This study |
| *S.* Typhi 120130191 | Clinical isolate of *S*. Typhi | *S*. Typhi isolate obtained at the SMC from a traveler returning from India and Thailand at 2012 |
| *S*. Typhi Δ*hilE* | Null deletion of *hilE* in *S*. Typhi 12013191 | This study |
| *S.* Sendai MZ1468 | Feb. 2008 outbreak strain 55-2461 (MZ1468) | Center for Disease Control, USA |
| *S*. Sendai Δ*hilE* | Null deletion of *hilE* in *S*. Sendai MZ1468 | This study |
| *S*. Paratyphi B SARA43 |  | [5] |
| *S*. Paratyphi B SARA44 |  | [5] |
| *S*. Enteritidis PT4 |  | SGSC4901 reference strain |
| *S*. Muenchen 180135033 |  | [6] |
| *S*. New port E2002725 |  | SGSC4910 reference strain |
| *S*. Infantis 119944 |  | [7] |
| *S*. 9,12: I,v:- | Clinical strain | lab collection |
| *S.* Agona 96109 | Clinical strain | lab collection |
| *S*. Bredenay SGSC 4931 |  | SGSC |
| *S*. Hadar 18 |  | SGSC |
| *S*. Heidelberg CVM30485 |  | SGSC |
| *S*. Hvittingfoss | Clinical strain | lab collection |
| *S*. Java | Clinical strain | lab collection |
| *S*. Mbandaka | Clinical strain | lab collection |
| *S*. Mississippi NVSL 10271 |  | SGSC |
| *S*. Montevideo SARB 30 |  | SGSC |
| *S*. Schwarzengrund CVM19633 |  | SGSC |
| *S*. Virchow | Clinical strain | lab collection |
| *Escherichia coli* strains | | |
| *E. coli* MC1022 | *E. coli* K-12 MC1022 cloning strain | lab collection |
| *E. coli* SM10 λ | Donor strain carrying transfer genes of broad host range; IncP-type plasmid RP4, Km^r^; *thi-1 thr leu tonA lacY supE recA*::*RP4-2-Tc*::*Mu pir* | George Church lab |
| **Plasmids** | | |
| pJA1 | Amp^R^, Tn10 transposon with a T7 promoter | George Church lab |
| pKD4 | Kan^R^ cassette template | [8] |
| pKD46 | Amp^R^, λ Red recombinase expression | [8] |
| pCP20 | Amp^R^, FLP recombinase expression | [8] |
| pMC1403 | Amp^R^, *lacZY* cloning vector | [9] |
| pMC1403/ *flhD::lacZ* | Amp^R^, promotor of *flhD* from *S.* Paratyphi A 45157 fused to *lacZ* cloned in pMC1403 | This study |
| pMC1403/ *flhB*_STM_*::lacZ* | Amp^R^, promotor of *flhB* from *S*. Typhimurium SL1344 fused to *lacZ* cloned in pMC1403. | This study |
| pMC1403/ *flhB*_SPA_*::lacZ* | Amp^R^, promotor of *flhB* from *S.* Paratyphi A 45157 fused to *lacZ* cloned in pMC1403. | This study |
| pMC1403/ *motA::lacZ* | Amp^R^, promotor of *motA* from *S.* Paratyphi A 45157 fused to *lacZ* cloned in pMC1403. | This study |
| pMC1403/ *fliC::lacZ* | Amp^R^, promotor of *fliC* from *S.* Paratyphi A 45157 fused to *lacZ* cloned in pMC1403. | This study |
| pWSK29 | Amp^R^, cloning vector | [10] |
| pWSK29::*hilE*_SPA_ | Amp^R^, *S*. Paratyphi 45157 *hilE* cloned into pWSK29 | This study |
| pWSK29::*hilE*_STM_ | Amp^R^, *S*. Typhimurium SL1344 *hilE* cloned into pWSK29 | This study |
| pWSK29::*hilD* | Amp^R^, *S*. Paratyphi 45157 *hilE* cloned into pWSK29 | This study |

REFERENCES

1. Gal-Mor O, Suez J, Elhadad D, Porwollik S, Leshem E, Valinsky L, et al. Molecular and cellular characterization of a Salmonella enterica serovar Paratyphi a outbreak strain and the human immune response to infection. Clin Vaccine Immunol. 2012;19(2):146-56. Epub 2011/12/23. doi: 10.1128/CVI.05468-11. PubMed PMID: 22190395; PubMed Central PMCID: PMC3272918.

2. Elhadad D, McClelland M, Rahav G, Gal-Mor O. Feverlike Temperature is a Virulence Regulatory Cue Controlling the Motility and Host Cell Entry of Typhoidal Salmonella. J Infect Dis. 2015;212(1):147-56. Epub 2014/12/11. doi: 10.1093/infdis/jiu663. PubMed PMID: 25492917.

3. Hoiseth SK, Stocker BA. Aromatic-dependent *Salmonella typhimurium* are non-virulent and effective as live vaccines. Nature. 1981;291(5812):238-9. Epub 1981/05/21. PubMed PMID: 7015147.

4. Elhadad D, Desai P, Rahav G, McClelland M, Gal-Mor O. Flagellin Is Required for Host Cell Invasion and Normal Salmonella Pathogenicity Island 1 Expression by Salmonella enterica Serovar Paratyphi A. Infect Immun. 2015;83(9):3355-68. Epub 2015/06/10. doi: 10.1128/IAI.00468-15. PubMed PMID: 26056383.

5. Achtman M, Hale J, Murphy RA, Boyd EF, Porwollik S. Population structures in the SARA and SARB reference collections of Salmonella enterica according to MLST, MLEE and microarray hybridization. Infect Genet Evol. 2013;16:314-25. Epub 2013/03/20. doi: 10.1016/j.meegid.2013.03.003. PubMed PMID: 23507027.

6. Cohen E, Kriger O, Amit S, Davidovich M, Rahav G, Gal-Mor O. The emergence of a multidrug resistant Salmonella Muenchen in Israel is associated with horizontal acquisition of the epidemic pESI plasmid. Clin Microbiol Infect. 2022;28(11):1499 e7- e14. Epub 2022/06/03. doi: 10.1016/j.cmi.2022.05.029. PubMed PMID: 35654317.

7. Cohen E, Rahav G, Gal-Mor O. Genome Sequence of an Emerging Salmonella enterica Serovar Infantis and Genomic Comparison with Other S. Infantis Strains. Genome Biol Evol. 2020;12(3):151-9. Epub 2020/03/08. doi: 10.1093/gbe/evaa048. PubMed PMID: 32145019; PubMed Central PMCID: PMCPMC7144548.

8. Datsenko KA, Wanner BL. One-step inactivation of chromosomal genes in Escherichia coli K-12 using PCR products. Proc Natl Acad Sci U S A. 2000;97(12):6640-5. Epub 2000/06/01. doi: 10.1073/pnas.120163297. PubMed PMID: 10829079; PubMed Central PMCID: PMCPMC18686.

9. Casadaban MJ, Chou J, Cohen SN. In vitro gene fusions that join an enzymatically active beta-galactosidase segment to amino-terminal fragments of exogenous proteins: Escherichia coli plasmid vectors for the detection and cloning of translational initiation signals. J Bacteriol. 1980;143(2):971-80. Epub 1980/08/01. PubMed PMID: 6162838; PubMed Central PMCID: PMC294402.

10. Wang RF, Kushner SR. Construction of versatile low-copy-number vectors for cloning, sequencing and gene expression in *Escherichia coli*. Gene. 1991;100:195-9. Epub 1991/04/01. PubMed PMID: 2055470.
