# Supplementary material for: HilE mediates motility thermoregulation in typhoidal Salmonella serovars at elevated physiological temperatures
Source: PLoS Pathog. 2025 Oct 16;21(10):e1013133. doi: 10.1371/journal.ppat.1013133 (PMC12561990; doi:10.1371/journal.ppat.1013133)
Supplement: S2 Table — (DOCX) [file ppat.1013133.s008.docx]

**S2 Table. Primers used in this study**.

| **Primer name** | **Sequence 5' to 3'** |
| --- | --- |
| Molecular biology and cloning^1^ | |
| hilE deletion F | aatctggtatacagagacaccaacgaaatggctggaaaatggaacGTGTAGGCTGGAGCTGCTTCG |
| hilE deletion R | ctgcgagtccgcaagcttgttttatcctcatcgccacagcgcctgCATATGAATATCCTCCTTA |
| hilE SacI F | ATATATGAGCTCaaaggaataagaattgtctatattc |
| hilE BamHI R | ATATATAGGATCCcaagcttgttttatcctcat |
| hilD deletion F | ttaaaataacatcaacaaagggataatatggaaaatgtaacctttGTGTAGGCTGGAGCTGCTTCG |
| hilD deletion R | ttacttaagtgacagatacaaaaaatgttaatggttcgccattttCATATGAATATCCTCCTTA |
| hilD SacI F | ATATATGAGCTCatatactgttagcgatgtctg |
| hilD BamHI R | ATATATAGGATCCttaatggttcgccatttttatg |
| dapB deletion F | acgcaatttttacaaaagagaatagctatgcatgaagcacaaatcGTGTAGGCTGGAGCTGCTTCG |
| dapB deletion R | caaccacatcacgaaggtatttagtcgctataatacatccagcccCATATGAATATCCTCCTTA |
| fliC deletion F | CAAGTTGTAATTGATAAGGAAAAGATCATGGCACAAGTCATTAATGTGTAGGCTGGAGCTGCTTCG |
| fliC deletion R | CCACGTGTCGGTGAATCAATCGCCGGATTAACGCAGATTAGAGAGCATATGAATATCCTCCTTA |
| CITRE_SPA_ F | TTGTTTCTCTCTGTTAAAATAACGCCAGGATAATAGATAACAGGCGTGTAGGCTGGAGCTGCTTCG |
| CITRE_SPA_ R | GCGTATGCTGTGACGAGATTGATTAATAACGTTTAATATTTAAGCCATATGAATATCCTCCTTA |
| CITRE_STM_ F | TTGTTTCTCTCTGTTAAAATAACGCTAGGATAATAGATAACAGGCGTGTAGGCTGGAGCTGCTTCG |
| CITRE_STM_ R | GCGTATGCTGTGACGAGATTAATTAATAACGTTTAATATTTAAGCCATATGAATATCCTCCTTA |
| flhDp SmaI F | AGAGAGCCCGGGGCCACATTAATGTGAAGGAC |
| flhDp BamHI R | GAGAGAGGATCCGATGTATGCATTGTTCCCAT |
| flhBp SmaI F | GAGACCCGGGGCTTTTTTGACCTGATGGCG C |
| flhBp BamHI R | GAGAGGATCCTTGTCGTCGTCGCTCTCTTC |
| fliCp SmaI F | GAGACCCGGGTAATGATGAAATTGAAGCCA |
| fliCp BamHI R | GAGAGGATCCGTATTAATGACTTGTGCCAT |
| motAp SmaI F | GAGACCCGGGAACCGAAACGGTGTGGACAA |
| motAp BamHI R | GAGAGGATCCACCAGGTAACCTAATAAGAT |
| Amplification of transposon insertion sites | |
| npt_A | TATCAGGACATAGCGTTGGC |
| npt_B | GATATTGCTGAAGAGCTTGGC |
| npt_C | TCTATCGCCTTCTTGACGAG |
| 1366-Arb-1A | ggccagcgagctaacgagacNNNNgatat |
| 1368-Arb-1B | ggccagcgagctaacgagac |
| qRT-PCR | |
| 16s F | GGTTAAGTCCCGCAACGAG |
| 16s R | CTTCTCTTTGTATGCGCCATTG |
| flhD F | TGTTCCGCCTCGGTATCAAC |
| flhD R | CGCGAATCCTGAGTCAAACG |
| flhB F | ATCAGCAGTGAGGTCAGTTC |
| flhB R | CGACGACAAAACAGAAGCCC |
| fliZ F | AAACATTTCCCACGATCTGC |
| fliZ R | CGGTAAAGGGGGATTTCTG |
| fliE F | AATTCACTCTGGGTGAGCCG |
| fliE R | CTTGTTGCGCACCTGAATCC |
| fliF F | CGATAGGCGACAGAATGGCT |
| fliF R | CACGCAATCCAATACCAGCG |
| fliK F | GTAAGCGTTTCATCCGTCGC |
| fliK R | ATCACGCTGGCCGATTTACA |
| fliL F | CACGAACGCTAAAGCGGAAC |
| fliL R | GTCTTTCAGACGCAGGGTCA |
| fliP F | TTGTTTTTGGCCTGTTGCGT |
| fliP R | GGCTGGTAAGCATCCACGTA |
| fliR F | CCATCGGTAGTAACCCGGTG |
| fliR R | CAGGTTGAGCGTAAGCAGGA |
| flgC-F | CGAGGCTGACATCGTGTTGA |
| flgC-R | CGCCAGCGTGATTGAAAGTC |
| flgF F | CAGTTGGACTACACCTCCCG |
| flgF R | CGGGTATATCCTTCAGCGCC |
| flgK F | TTCTCAAGCCGGTCAGCAAT |
| flgK R | TGCCTGACCATTGCGGTTAT |
| flgM F | CCTTTGAAACCCGTTAGCAC |
| flgM R | GCCGTTTTTAATGCTTCGAC |
| fliS F | CCTGCAGTAAACGGCGAATC |
| fliS R | GTTGTCGCGAAAGGTGAAGC |
| fliC F | AAGAGAGGACGTTTTGCGG |
| fliC R | CGAAGATTCCGACTACGCG |
| fljB F | TTTTACCGTCTACGCCACCC |
| fljB R | TACGATGAAGCGACAGGAGC |
| motA F | TGCCGTGGAATTTGGTCGTA |
| motA R | CTGCTGCTGGTTTGGGTTTC |
| cheA F | ACTGTCGGCAACTCTGGATG |
| cheA R | GCGCTACCTCGGTTTTCTCT |
| cheB F | GCACATGTCGGATAGCTTC |
| cheB R | AGATGATCGCCGAAAAAGTG |
| tsr F | ACAGTTCCTACAACCAGGCG |
| tsr R | ATCAGACGATTCATCGGGGC |
| aer F | AGACGAATATCCTGGCGCTG |
| aer R | CCTGACTGCACCTTTGTTGC |

^1^ Restriction sites introduced into the primers are underlined
