# Supplementary material for: HilE mediates motility thermoregulation in typhoidal Salmonella serovars at elevated physiological temperatures
Source: PLoS Pathog. 2025 Oct 16;21(10):e1013133. doi: 10.1371/journal.ppat.1013133 (PMC12561990; doi:10.1371/journal.ppat.1013133)
Supplement: Attachment — Submitted filename: Response to Reviewers 28_07_25.pdf [file ppat.1013133.s011.pdf]

SHEBA Tel HaShomer City of Health

GAL-MOR LAB THE INFECTIOUS DISEASES RESEARCH LABORATORY

TEL AVIV UNIVERSITY אוניברסיטת תל אביב

Saturday, August 02, 2025

Dear *PLOS Pathogens* Editor,

We would like to thank the editor and the two anonymous reviewers who critically evaluated manuscript PPATHOGENS-D-25-00929 titled "**HilE mediates motility thermoregulation in typhoidal *Salmonella* serovars at elevated physiological temperatures**". We found their comments constructive and helpful in revising the manuscript. Based on the reviewer suggestions we have performed additional experiments, revised the manuscript structure, changed data presentation to improve clearance, and made multiple edits. We believe that these revisions significantly improved the manuscript. Please find below a point-to-point response (in red) to each one of the reviewer comments.

Part I - Summary

Reviewer #1: This manuscript describes in an over complicated fashion the mechanistic explanation for a previously recognised thermoregulation of flagellar gene expression in Salmonella serovars. Embedded deep in this dialogue is a further interesting observation with respect to the impact the thermoregulation has upon macrophage uptake. This second observation is in some essence more important than defining the role HilE plays in controlling flagellar gene expression. It is the combination of these two points that I can understand why the authors have submitted this paper. However, from my point of view as a reviewer there is substantial work to be done on the data presentation to allow a reader to easily follow their rationale for the experiments performed and the analysis of their data.

I am, however, confident that the authors can recognise the constructive approach of this review and in doing so generate a nice narrative of two/three observations for Salmonella as a species. They show that HilE controls thermoregulation of S. Paratyphi, not all serovars exhibit this phenotype and that it has a marked impact on macrophage uptake. Please consider restructuring the results based on this order.

Response: We thank the reviewer for their meticulous evaluation and pointing out the manuscript strengths and suggestions for improvement. We have now modified the order of the results section to improve the manuscript's flow and revised much of the data presentation to enhance clearance and data interpretation.

Reviewer #2: Shem-Tov and Gal-Mor identified in their manuscript the SPI-1 repressor HilE as a negative regulator of motility at elevated temperatures for typhoidal Salmonella serovars. While the group described the inhibition of bacterial motility at 42°C for specific for typhoidal serovars in an earlier manuscript, they reproduced their finding in the present manuscript at 40°C more closely resembling enteric fever in patients. To elucidate the mechanism behind, the group designed a chromogenic reporter system allowing for high throughput screening of flagellar gene induction. This reporter system was combined with a dense transposon screen to identify genes responsible for the attenuated motility at febrile temperatures in a multi-stage screening setup. Here, genes involved in lysine production (yffB, dapBE) and SPI-1 related genes (invG, sipD, hilE) were identified. Functional characterization of knockout mutants identified HilE to be responsible for the observed phenotype. Since HilE is described as a negative regulator of the SPI-1 master regulator HilD, the group also tested hilD deletion mutants in reporter, motiliy and qPCR assays which demonstrated the HilE effect to be mediated via HilD. HilD probably acts via transcriptional activation of flagellar master regulator FlhDC. Moreover, the group tested the uptake of S. Paratyphi A (*S. Paratyphi A*) in human THP-1 macrophages. Absence of HilE led to increased uptake of *S. Paratyphi A* at 40°C. Finally they tested different other typhoidal and non-typhoidal serovars (NTS) for the motility phenotype at 40°C. Interestingly, only typhoidal Salmonella with the exception of S. Paratyphi B showed the reduced motility at 40°C. Deletion of hilE in the typhoidal serovars *S. Paratyphi A*, Typhi and Sendai increased motility at 40°C.
The manuscript is clearly and well written making the argumentation of the authors comprehensible. However, although identifying the HilE-HilD axis to be involved in thermoregulation of motility, an actual temperature-dependent mechanism remains elusive. In their working model, HilE should have an increased affinity for HilD in typhoidal Salmonella (except SPB) compared to NTS (and SPB). Can this be explained on the molecular level and ideally addressed experimentally?

Response: We appreciate this comprehensive and detailed summary by the reviewer. We have conducted further suggested experiments with an effort to understand the differences of HilE and HilD between *S*. Typhimurium and *S*. Paratyphi A. These efforts included swapping experiments, in which HilE from *S*. Typhimurium was expressed in *S*. Paratyphi A and *vice versa*. These new experiments now presented in Figures S5 and S6 point out to additional regulatory factor involved in the described motility thermoregulation phenotype, possibly the histone-like nucleoid structuring protein, HNS.

Although I can follow the argumentation of the authors to include the infection data of THP-1 macrophages (Fig. 6, systemic spread in enteric fever), it still feels a little disconnected from the rest of the manuscript.

Response: We agreed with this point. In the revised manuscript, we expanded this analysis to include additional *S*. Typhimurium and other typhoidal (Typhi and Sendai) strains. We also

reorganized the order of the results section and now report these findings last, in a way that improves the internal flow and the thematic connection of this section to the rest of the results.

Part II – Major Issues: Key Experiments Required for Acceptance

Reviewer #1:

No further experiments are necessary. However, The data in figure 6 would benefit similar mutants in other Salmonella serovars being tested and included in this analysis. Possibly something to think of for the future maybe.

Response: We accepted the reviewer's suggestion and expanded Figure 6 (now numbered Figure 7) to include *S*. Typhimurium, Typhi and Sendai WT and Δ*hilE* mutant backgrounds.

Reviewer #2:

1. Can the authors demonstrate the temperature-dependent difference of the HilD-HilE interaction using co-IP or suitable reporter systems (e.g. as shown in Grenz et al. J. Bacteriol 2018 https://doi.org/10.1128/jb.00750-17).

2. Are there differences in the sequences of HilD and/or HilE which might explain this different behavior? If differences exist, do typhoidal Salmonella separate from NTS (and SPB) in unsupervised clustering of sequence alignments? Can critical amino acids be identified based on the known structure of the HilD-HilE complex?

3. Could there be temperature- and serovar dependent differences in HilE/D protein stability?

4. How does *S. Typhimurium* behave in the motility assay expressing HilE and/or HilD of *S. Paratyphi A* and vice versa?

Response to points 1-4: HilD is identical in *S*. Typhimurium and *S*. Paratyphi A (New Fig. S5A). HilE diverse in three amino acids between its orthologues in *S.* Paratyphi A and *S.* Typhimurium. To know if these variations are responsible for the distinct motility thermoregulation, we expressed the *S.* Typhimurium version of HilE in *S.* Paratyphi A and *vice versa*. As shown in Fig. S5C, this protein swapping did not affect the basic thermoregulation that was still observed. We concluded that although HilE mediates motility thermoregulation in *S.* Paratyphi A, it is required but not sufficient to the motility thermoregulation in this pathogen. In agreement with this hypothesis, we identified a new cis regulatory element in the p5 promoter of *flhD* that is also involved in *S.* Paratyphi A motility thermoregulation. Promoter analysis predicted that this 49-bp region contains two H-NS binding sites, which is also known to act as a transcriptional repressor [PMID: 19207739] and as a HilD antagonist [PMID: 25135218]. Therefore, we speculate that additional regulatory factor

(possibly H-NS), is also required in conjunction with HilE to orchestrate motility thermoregulation in *S*. Paratyphi A. We have now included this new information in new Figures S5 and S6 and in the Discussion section in lines 567-581 in the Marked up revision.

5. Based on their findings, there should be a major impact of SPI-1 T3SS expression at 40°C vs. 37°C in typhoidal Salmonella. Did the authors also test invasion of non-phagocytic cells under these conditions?

Response: Yes, this is a good point because HilE primarily regulates SPI-1. Invasion into non-phagocytic cells under elevated temperature has been studied and reported by our group a few years ago (PMID: 25492917).

6. Although the group previously showed that entry in macrophages depends on functional flagella, it would be interesting to dissect the influence of motility/chemotaxis (near surface swimming) from flagella-mediated adhesion. Here, the authors could compare infection with (as in the present manuscript) and without mild centrifugation.

Response: we accepted this suggestion and compared *S. Paratyphi A* uptake with and without centrifugation. As can be seen in the new Fig. S4, adding a mild centrifugation step indeed increased the overall uptake of *S.* Paratyphi A by THP-1 macrophages and even allowed detectable entry of the *fliC* mutant. However, centrifugation did not change much the enhanced uptake of the ∆*hilE* mutant or the effect of the temperature, suggesting that the contribution of HilE goes behind merely the improvement in bacteria motility and is likely connected to additional roles of the flagella in host cell interactions, such as flagella-mediated adherence [PMID: 25590430; PMID: 26056383]. We have now added this information in lines 436-441 in the Marked up revision.

7. I'm wondering about the expression of hilE and hilD in O/N cultures. Depending on the conditions (aeration, NaCl content) SPI-1 expression could be rather low. Did the authors check with RT-qPCR expression of these genes (and flagellar genes)?

Response: Overnight cultures were used only for the macrophage uptake experiments. In these experiments, we wash and dilute *Salmonella* O/N cultures in RPMI medium and infect the cells for 2 h before we lyse and determine uptake. Although expression of SPI-1 and motility genes is indeed much lower at the stationary phase, dilution and incubation of S. Typhimurium and S. Paratyphi A in RMPI, even for an hour, induces their expression significantly (not shown in the manuscript).

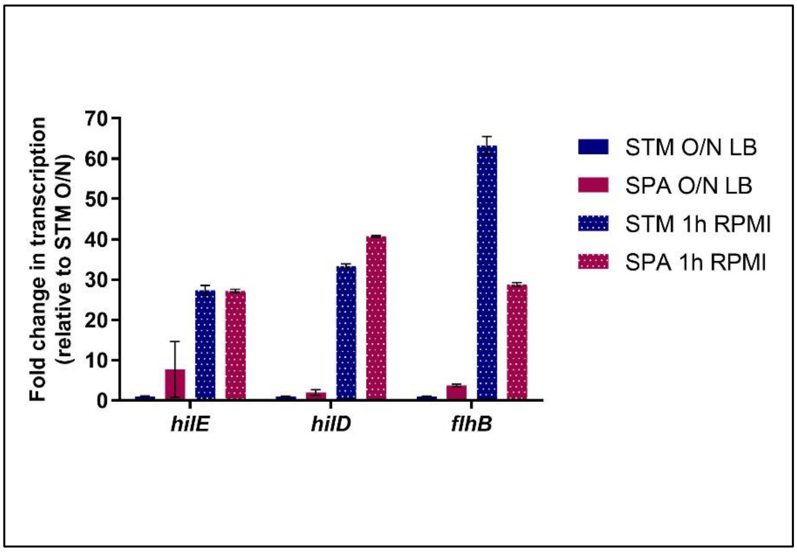

Part III – Minor Issues

Reviewer #1:

1. Please refrain from using *S. Typhimurium*, *S. Paratyphi A* etc it is not difficult to write S. Typhimuirum and S. Paratyphi A in its place and really helps the reader follow which serovar you are using.

Response: We have modified the serovars abbreviation to their full scientific names throughout the manuscript.

2. Please rename all mutants as ΔhilE, ΔhilD etc etc instead of hilE and hilD, this will then conform with standard genetic practice and allow the reader to differentiate between you discussing a gene, its overexpression, its expression and its deletion.

Response: Suggestion accepted and the jargon of all null deletion strains was modified accordingly.

3. Please place the supplementary figures into the supplementary document to go along with their legends. Having a figure with no legend in such material is pointless.

Response: Thank you for pointing this out. The Supplementary Material was organized as suggested.

4. Figure Y-axis: I had a hard time understanding your y-axis choice for many of the graphs and thus the interpretation of most of the data. It would be appreciated if you follow the rules of using a uniform axis (-2 to +2) and not have the X-axis cross the Y-axis at 1 when in essence these are normal bar charts. For example, the data in figure 1 suggests a very strong downregulation of motility when in reality S. Paratyphi A is MOTILE at 40°C but I agree is reduced compared to S. Typhimurium. As a result, the data in A and B does not really correlate with the phenotype observed which is a 3-fold

reduction in motility as assayed by the semi-solid agar swarming assay. This will be a recurring theme that I am concerned that your expression data at times does not correlate well with the observed phenotype.In summary, after addressing the reviewer comments, adding new analyses and data, and revising the text, we hope you will find the manuscript appropriate for publication in *Virulence*.

Response: We do understand that the titles and Y-axis organization at some of the figures was not clear enough. In the revised version we have modified the data presentation and/ or Y-axis title of figures 1A, 3C and D, 4A and D, 5D, 6A and C, and 7A. We believe that the charts are now more understandable.

5. Figure 3: You do not need to convince a microbiologist/microbial geneticist that the colonies you have isolated were blue as shown in Fig 3A&B. If you really want to include this data place it in the supplementary data and edit out the text on page 10 lines 195 to 205.

Response: We appreciate and understand why the reviewer has raised this comment. However, we think that there is a value in presenting panels A and B as it gives the readers a better sense about the distribution of the thermoregulatory mutants among the entire Tn library and visually demonstrates how different mutants resulted in varying expression of the reporter system, in relation to the WT strain. Considering the centrality of the genetic screen in our report, we have chosen to include these images in Fig. 3.

6. Page 10 Genetics: Please justify why the numbers of mutants that are analysed keeps changing or present the data for all 23. You can have a clear statement that for this study the focus was HilE at the end of this genetic section, but please stay consistent. Please also make sure you numbering is consistent in Figure 3 you have these as 3-3 3-2 13-3 etc etc but in table 1 they are 3-15 3-19 etc etc which way around are they supposed to be? You must have sequence data for all of them so why are you only showing these.

Response: The number of the Tn mutant contain the screening cycle (1-3) and the number of the isolated mutants (1-23), we have now edited Table 1 and the relevant figure for consistency. As for the number of the analyzed clones, the varying numbers represents the different filtration steps (blue colonies at first plating of the library, stable blue appearance following replating, quantitative β-galactosidase assay, and finally change in motility at 40°C. Clones that have not passed the filtration criteria were excluded from the next screening step. This approach is explained in lines 208-223 in the Marked up revision.

7. Figure 3D, 4D, 5D, 7A and 7C. You are not presenting ‘relative motility data’ in these graphs. Furthermore, these graphs specifically relate back to point 3 regarding the irregular use of y-axis

scales, the over exaggerated axis scale does not represent the true impact of these genetic manipulations. I could not generate from the methods or my own assessment of your motility data shown in 3C how you came to these numbers. It is not simply the radius or diameter of the swarms divided by each other, and please do not use the argument that the 3D image is just an example, you are showing data that should in some context correlate to the quantification. I tried to do this using a ruler from your figures. For example, WT 37°C 41 mm 40°C 28 mm which equates to 0.68 or a 1.5 fold reduction and not ~0.3. Your data is robust and in every case is justified, just please consider presenting the reduction in an easier understandable format. Even if it is the quantified means placed next to each other on a bar chart without any convoluted further calculations.

Response: Thank you for this point. We have changed the title of the Y-axis of relevant motility data to indicate “Motility at 40° relative to 37°C” which is more accurate. Swimming motility was analyzed at two kind of plates (using 90 mm and 140 mm plates). The time of incubation, the radius of the plates used, and even if we spotted several strains together on one plate (e.g. Fig. 3E) or one strain per plate (e.g. Fig. 1C), were all affected the swimming motility rate, **but not the overall impact on the phenotype**. We understood that it can be confusing and have now presented matched data sets to be more consistent between the chart and image.

8. Figure 4: The Ponceau S staining is not necessary in any of the figures or again if you feel it is put it in supplementary data. Furthermore, once you have defined your motility quantification method and presentation style you do not need to always show the motility swarms. In this instance as well as other places the motility swarm data is not correlating well with expression or quantification. Figure 4A please state what scale format is being used on the y-axis or again just use a linear scale so the reader can see how small some of the values are.

Response: We can relate to this reviser’s comment, but feel that we need to provide a reliable loading control in association with the Western blots. We have accepted the comment about Fig. 4A, changed the presentation on the Y-axis and better matched the presented data sets in Fig. 4D with the selected image (please see our response to this comment at the point above).

9. Page 15 297-318: You could discuss this data in a much simpler manner. You also overlook either purposefully or non-intentionally the impact of ΔhilE at 37°C.

Response: This section was edited and revised to address the reviewer’s comments and is now explained in lines 325-330 in the Marked up revision.

10. Figure 6 please reassess your fold reduction statements or at least make it clearer what is being compared. For example, ΔhilE @ 40°C is ~ 150% ΔhilE @ 37°C is ~75% that’s a 2-fold difference not a 3-fold difference (page 17 line 358).

Response: Fig. 6 (now presented as Fig. 7) was revised to present absolute uptake data (and not relative uptake) to make the comparison clearer. Additionally, we edited the relevant text in lines 407-429 in the Marked up revision.

11. Figure legends: There is too much methodology hidden within the legends. If this is not in your methods section add it to the methods. If it is in the methods do not repeat it here. Please also explain what is meant in the methods by four biological repeats in one representative, out of two independent experiments is this n= 4 or n = 8?

Response: We agreed with the reviewer that the Figure legends were overloaded with technical details and revised all of them to improve their flow and conciseness.

12. Figure 7 Legend: Why were different strains measured at different times and why is this important enough for you to mention it? As you have mentioned it you really need to justify why in the main text or in the legend.

Response: The time of motility swimming is important because these serovars demonstrated varying motility speed. In addition, as mentioned above, the incubation time affected the motility radius, that may vary between experiments and therefore is important to be mentioned.

13. Page 23 line 478 This is the first EVER mention of SARA 43 and SARA 44. If there is a reason you used both, this needs justifying and possible highlighting in the results section when narrating through figure 7.

Response: Yes, in Fig. 6 we expanded the examined serovars and included more typhoidal and NTS serovars that were not tested in previous analyses. SARA 43 and SARA 44 were included as two *S*. Paratyphi B strains. Please note that all used strains and their sources are listed in Table S1.

14. Discussion: I liked the discussion and how it correlates to how Salmonella as species interacts with us. I did not look at figure 8 although I did note it was nicely presented.

Response: Thank you very much for this nice comment. Considering that motility was shown by many groups as an important virulence-associated phenotype, we certainly believe that the reported findings have a physiological and clinical relevance.

Reviewer #2:

Minor point: Instead of using bars only for the plots, it is generally recommended to show in addition the individual data points making up that bar.

Response: The reviewer is right. All the charts were modified to show the individual data points. Thank you for this comment.

In summary, after addressing the reviewer comments, adding new analyses and data, and revising the text, we hope you will find the manuscript appropriate for publication in *PLOS Pathogens*.

Sincerely,

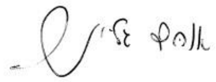

Ohad Gal-Mor
Head, the Infectious Diseases Research Laboratory, Sheba Medical Center, Tel-Hashomer 5262100, Israel
Professor, Department of Clinical Microbiology and Immunology, Gray Faculty of Medical and Health Sciences, Tel-Aviv University
Chair, The Biomedical Academic Research institute (BARI) at the Sheba Medical Center
Director, the Nehemia Rubin Program for Excellence in Biomedical Research (Telem Program)
Tel: (972)-3-5307993 (Office); (972)-3-5303458 (Lab)
E-mail: Ohad.Gal-Mor@sheba.health.gov.il
Website: https://www.gal-mor-lab.com/
