# Supplementary material for: HilE mediates motility thermoregulation in typhoidal Salmonella serovars at elevated physiological temperatures
Source: PLoS Pathog. 2025 Oct 16;21(10):e1013133. doi: 10.1371/journal.ppat.1013133 (PMC12561990; doi:10.1371/journal.ppat.1013133)
Supplement: Attachment — Submitted filename: Response to Reviewers R2.pdf [file ppat.1013133.s012.pdf]

SHEBA Tel HaShomer City of Health

GAL-MOR LAB THE INFECTIOUS DISEASES RESEARCH LABORATORY

TEL AVIV UNIVERSITY אוניברסיטת תל אביב

**Wednesday, September 17, 2025**

Dear *PLOS Pathogens* Editors,

We would like to thank the editors and the reviewers for their evaluation of our revised manuscript PPATHOGENS-D-25-00929R1 titled "**HilE mediates motility thermoregulation in typhoidal *Salmonella* serovars at elevated physiological temperatures**". We are pleased that both reviewers and the editors were positive and appreciated the thorough revision made.

As asked by the editor and Reviewer 1, we have changed the charts in Figures 3C and D, 4D, 5D, 6A and C, S2A and C, and S5D to show the **fold change in motility** at 40°C relative 37°C instead of showing the **motility ratio between 40°C and 37°C**. These modifications also involved change in X-axis crossing at 0, as Reviewer 1 asked.

Nonetheless, it will be difficult and misleading to present the data in Figure 1B in the same way. The reason for that is that many motility proteins were basically undetected using a sensitive mass spectrometry analysis at 40°C (zero abundance), but were detected at 37°C. Presenting fold change in protein expression will require to show values that are close to infinity (∞) on the graph. In contrast, showing the **protein abundance ratio** between 40°C and 37°C is zero and possible to present on the graph with intuitive interpretation by the reader. To increase clarity, we changed the Y-axis's label in Fig. 1B to indicate "Ratio of protein abundance at 40°C to 37°C ."

Minor changes in the figure legends were also made in compliance with the above graph modifications. No other changes made according to the editor request.

I hope that with these changes, the manuscript is now acceptable for publication in *PLOS Pathogens*.

Sincerely,

Ohad Gal-Mor
Head, the Infectious Diseases Research Laboratory, Sheba Medical Center, Tel-Hashomer 5262100, Israel

Professor, Department of Clinical Microbiology and Immunology, Gray Faculty of Medical and Health Sciences, Tel-Aviv University
Chair, The Biomedical Academic Research institute (BARI) at the Sheba Medical Center
Director, the Nehemia Rubin Program for Excellence in Biomedical Research (Telem Program)
Tel: (972)-3-5307993 (Office); (972)-3-5303458 (Lab)
E-mail: Ohad.Gal-Mor@sheba.health.gov.il
Website: https://www.gal-mor-lab.com/
